# Editorial: Role of Inner Ear in Self and Environment Perception

**DOI:** 10.3389/fneur.2020.00022

**Published:** 2020-02-20

**Authors:** Christophe Lopez, Michel Toupet, Christian van Nechel, Alexis Bozorg Grayeli

**Affiliations:** ^1^Aix-Marseille University, CNRS, LNSC, Marseille, France; ^2^Centre d'Explorations Fonctionnelles Otoneurologiques, Paris, France; ^3^Institut de Recherche Oto-Neurologique (IRON), Paris, France; ^4^Clinique des Vertiges, Brussels, Belgium; ^5^Otolaryngology Department, Dijon University Hospital, Université Bourgogne-Franche Comté, Dijon, France; ^6^Electronic, Image and Computer Research Laboratory, Le2i, Dijon, France

**Keywords:** self-motion, vestibular system, vestibular rehabilitation, postural control, visual vertical, depersonalization and derealization, dizziness and vertigo, nystagmus

Otoneurology and vestibular neuroscience recently advanced with a better understanding of the vestibular contributions to perceptual and cognitive functions, reaching far beyond balance, and eye-movement control. Pioneering clinical observations established connections between dizziness and altered sense of self, distortions of the body schema, and symptoms resembling depersonalization and derealization (1, 2). However, studies in large samples of patients with dizziness have only recently validated the assumption that the vestibular system is the main contributor to the bodily self (3, 4). Recent epidemiological studies have also linked vestibular disorders to cognitive deficits. For example, a survey in over 20,000 adults established that individuals reporting vestibular vertigo had an eight-fold increase in the odds of reporting impaired memory and attention, limiting their activities (5). Another survey conducted in adults over 60 years revealed that vestibular impairment partially mediated the association between age and cognitive impairment. It was estimated that vestibular impairment mediates 14.3% of the effects of age on cognition and that it accelerates cognitive decline by 5 years in a visuo-spatial test (6). These studies raise the necessity to investigate more carefully the effects of vestibular impairment in dementia and several psychiatric disorders (7, 8).

Research in this area benefited from a better delineation of the human vestibular cortex. Functional MRI (fMRI) studies and meta-analyses of neuroimaging data have revealed that the cortical vestibular network is centered on the operculo-insular/retroinsular cortex (9) and that vestibular inputs also project to the temporo-parietal junction, cingulate cortex, somatosensory cortex, posterior parietal cortex, hippocampus, and frontal eye fields (10). These widespread vestibular projections to the brain were recently confirmed in whole-brain functional mapping in rodents using fMRI, local field potentials, and optogenetics (11, 12). We note that recent descriptions of functional connectivity, metabolic, and morphological brain alterations in peripheral vestibular disorders or chronic subjective dizziness [e.g., (13–15)] offer the possibility to evaluate, in a non-invasive manner, how various vestibular rehabilitation methods and drugs can improve brain plasticity. Finally, recent fMRI studies revealed the influence of cognition, emotion, and personality traits (such as neuroticism and introversion) on vestibular information processing [e.g., (16, 17)]. These observations suggest an expansion of the vestibular brain network into dimensions of emotion processing, mental health, and social cognition.

This Research Topic collection includes 17 articles combining contributions from authors with a large range of expertise in medicine and basic science, including neurology, otorhinolaryngology, neurophysiology, physiotherapy, neuropsychology, cognitive neuroscience, and bioengineering. This initiative brought together authors affiliated to institutions from six continents and 17 countries (Belgium, Bulgaria, Canada, France, Germany, Iran, Italy, Ivory Coast, Mexico, Netherlands, New Zealand, Russia, Spain, Switzerland, Taiwan, the United Kingdom, and the United States), showing the worldwide interest in advancing research in otoneurology, vestibular physiology, and vestibular cognition.

Altogether, contributions from this Research Topic highlight recent discoveries in otoneurology regarding (1) upright perception; (2) self-motion perception and balance control; (3) self and own-body cognition; and (4) vestibular physiopathology, testing, and rehabilitation.

## Upright Perception: From Neuroimaging to Clinical Assessment

An important function of the vestibular system is the perception of the visual vertical (VV), which contributes not only to own-body orientation and postural control but also to the judgment of orientation and even stability of objects in the environment. Psychophysical and clinical studies showed that vestibular signals are integrated with visual, tactile, proprioceptive, and interoceptive signals for an accurate VV perception, probably following Bayesian rules (18). In line with a developmental and multisensory perspective, Cuturi and Gori document visual, haptic, and visuo-haptic vertical perception in children and young adults tilted 90° to their side. Bayesian modeling reveals a lack of multisensory integration in children for the bimodal task.

Dieterich and Brandt offer a comprehensive overview of the pathways underpinning VV perception from the inner ear to the cerebral cortex, describing the consequences of lesions of various structures along these pathways. Using fMRI in healthy adults, Saj et al. show that VV perception involves the temporo-occipital and parieto-occipital networks (including the cuneus, lingual gyrus, and precuneus), together with areas in the cerebellum and brainstem, in accordance with observations in neurological patients (19, 20). Similar networks have been involved in navigation, balance control, and body representations, suggesting overlaps between these functions and VV perception.

Although VV perception is a widely used test of peripheral vestibular functions, it is not commonly used in neurological conditions such as vestibular migraine. Winnick et al. found that patients with vestibular migraine, but normal vestibular function, show abnormal VV when their head is tilted to the right (as if they were further tilted), whereas their VV is normal with their head upright or tilted to the left. This is corroborated by symptoms (tilting, pulling, and rotation) reported by the same patients mainly to their right side. These observations indicate abnormal multisensory integration for VV perception in vestibular migraine, consistent with abnormal self-motion perception recently measured in vestibular migraine (21).

## Self-Motion Perception and Balance Control: From Psychophysics to Clinical Assessment

Self-motion perception also involves several motion sensors and multisensory brain areas. Its exploration is crucial to understand self–environment interactions (22). Britton and Arshad thoroughly review human and animal studies of the relations between sensory signals and processing centers, as well as their impact on cognitive functions, such as navigation, spatial awareness, and emotions. Although many connections are to be explored, the authors present the system from an interesting perspective of integrative physiology. Kolev analyzed interindividual differences in the perception of self-motion vs. environment-motion perception after a rotatory chair stimulus. The author reports a significant effect of insinuation and sex on motion perception, opening insights into the mechanisms of motion sickness.

Two studies describe sensorimotor adaptations underpinning self-motion perception and balance control in patients with a bilateral vestibular failure (BVF). Lacour et al. show that BVF patients improve balance during fixation of a target, as well as during pursuit of a target with slow eye movements and saccades. They propose that BVF patients may use more efficiently proprioception from extraocular muscles or efference copy from the eye motor command than healthy controls. Guigou et al. explore the effect of a rotating sound on balance. A rotating sound destabilizes patients with BVF and with bilateral cochlear implants, indicating that hearing can also be used for postural control in patients with sensory deficits.

Magnetic vestibular stimulation in an MRI scanner can also evoke sensations of self-motion and horizontal nystagmus (23). Ward et al. show that visual information and continuous head rotations in the magnetic field do not influence the set-point adaptation (i.e., mechanisms attempting to inhibit unwanted nystagmus) of the nystagmus. This is in contrast with the well-known effect of vision on adaptation of the vestibulo-ocular reflex (VOR).

## Self and Own-Body Cognition

Three original research articles highlight the increasingly recognized role of the vestibular system in the sense of self and in own-body cognition. In a large sample of 319 patients with chronic dizziness, Toupet et al. show increased symptoms of depersonalization–derealization with increased levels of anxiety and depression. In addition, they show that depersonalization–derealization is associated with higher visual and vestibular hypersensitivity, migraine, and motion sickness. Aranda-Moreno et al. report that caloric vestibular stimulation and unilateral centrifugation both decreased phantom limb pain and symptoms of depersonalization–derealization reported by amputees. In an original study conducted in healthy participants, Thür et al. adapt the full-body illusion, a visuo-tactile illusion of self-identification with a virtual character (24), to include sensory conflicts between gravitational and visual cues. Their results suggest a mutual interaction of graviceptive and other sensory signals and the individual's weighting style in defining the sense of self.

## Advances in Vestibular Physiopathology, Testing, and Rehabilitation

Vestibular dysfunctions have been associated with anxiety and hyperactivity (25), premature cognitive decline, or Alzheimer's disease (26). Other neurological disorders may also be partly related to vestibular disorders. Here, Smith summarizes recent research in humans and animals, establishing connections between the vestibular system and Parkinson's disease.

Standard otoneurological evaluation of the peripheral vestibular system now includes the video head impulse test, which is useful to detect vestibular hypofunction by testing the VOR during high-velocity head rotations (27). In a study conducted in BVF patients, Van Nechel et al. analyze the factors influencing the catch-up saccades (visible target vs. in darkness with imaginary target), which are generated to compensate deficient VOR. The authors propose that visual signals are the main trigger and parametric determinant of the catch-up saccades and that a target is necessary in most cases to generate catch-up saccades.

In addition to testing reflexive eye movements, Dupuits et al. propose that vestibular evaluation can benefit from systematic measure of vestibular perception thresholds using whole-body motion platforms, as done in standard psychophysics experiments in healthy participants (28). They measured vestibular perceptual thresholds using a hydraulic platform in the dark delivering six translations and six rotations/tilts.

In a perspective of vestibular rehabilitation, Sadeghi et al. show that unidirectional whole-body rotations toward the side of the vestibular deficit decreased VOR asymmetry even 10 min after one rehabilitation session. In a long-term study, VOR asymmetry decreased to reach normal values during the first two sessions in most patients. Finally, Idoux et al. developed a model to evoke motion sickness in mice and tested how scopolamine (a muscarinic antagonist) can prevent motion sickness at the behavioral and cellular levels. They report that both motion sickness and scopolamine decrease VOR efficacy, which might be a protective mechanism to prevent later occurrences of motion sickness. The authors set the basis for studies of motion sickness in rodents and offer translational perspectives for improving treatment of motion sickness in humans.

## Author Contributions

CL and AB wrote the manuscript. CL, AB, MT, and CN revised the manuscript.

### Conflict of Interest

The authors declare that the research was conducted in the absence of any commercial or financial relationships that could be construed as a potential conflict of interest.
